# The Relevance of Crystal Forms in the Pharmaceutical Field: Sword of Damocles or Innovation Tools?

**DOI:** 10.3390/ijms23169013

**Published:** 2022-08-12

**Authors:** Dario Braga, Lucia Casali, Fabrizia Grepioni

**Affiliations:** Department of Chemistry G. Ciamician, University of Bologna, Via Selmi 2, 40126 Bologna, Italy

**Keywords:** crystal polymorphism, hydrates, co-crystals of active pharmaceuticals

## Abstract

This review is aimed to provide to an “educated but non-expert” readership and an overview of the scientific, commercial, and ethical importance of investigating the crystalline forms (polymorphs, hydrates, and co-crystals) of active pharmaceutical ingredients (API). The existence of multiple crystal forms of an API is relevant not only for the selection of the best solid material to carry through the various stages of drug development, including the choice of dosage and of excipients suitable for drug development and marketing, but also in terms of intellectual property protection and/or extension. This is because the physico-chemical properties, such as solubility, dissolution rate, thermal stability, processability, etc., of the solid API may depend, sometimes dramatically, on the crystal form, with important implications on the drug’s ultimate efficacy. This review will recount how the scientific community and the pharmaceutical industry learned from the catastrophic consequences of the appearance of new, more stable, and unsuspected crystal forms. The relevant aspects of hydrates, the most common pharmaceutical solid solvates, and of co-crystals, the association of two or more solid components in the same crystalline materials, will also be discussed. Examples will be provided of how to tackle multiple crystal forms with screening protocols and theoretical approaches, and ultimately how to turn into discovery and innovation the purposed preparation of new crystalline forms of an API.

## 1. Introduction

Crystal polymorphism has been known and studied since the early days of solid-state chemistry and crystallography [1,2], but it is only in the recent past that it has emerged as a strategic research area involving the use of molecular crystalline materials (pharmaceuticals, nutraceuticals, fertilizers, pigments, high-energy materials, etc.) [3,4,5,6].

Although the unexpected appearance of a new crystal form of a known active principle is often a threat for an API on the market (see below), it is also true that the urge for a careful pre-screening and form selection is a potent stimulus for research in various areas, and provides opportunities for innovation and new discoveries, especially in the burgeoning subfield of molecular co-crystals. This latter class of crystalline compounds is proving particularly apt to innovation and the development of new drugs and/or of new formulations of old ones [7,8]. 

In order to help the reader to put the current academic and industrial interest on crystal forms into a wide perspective, this review will move from the early days awareness of the importance of searching for crystal forms to the current impact and consequences for the scientific community and for the industrial sector of the discovery of polymorphs, solvates, and co-crystals of APIs.

## 2. Polymorphism: The Awareness

The existence of a substance in more than one crystal form has been known since 1822 [1], but we have to wait until 1962 for Walter McCrone, unanimously recognized as the father of this field of research, to provide the definition of a polymorph as “a solid crystalline phase of a given compound resulting from the possibility of at least two crystalline arrangements of the molecules of that compound in the solid state …every compound has different polymorphic forms…” He also opinioned that “the number of forms known for a given compound is proportional to the time and money spent in research on that compound” [2]. This “prediction” was published in 1962, but it took more than three decades before the phenomenon of polymorphism would hit the pharmaceutical field in a rather shocking way (see below the Section 5).

In addition to polymorphs, i.e., crystals having the same chemical composition but different structures, the term “crystal forms” nowadays encompasses not only the association of the molecule of interest with solvents (solvates), but also the association with molecules that form solids at room temperature (co-crystals) or with salts (ionic co-crystals). It is important to stress that all these crystal forms can be polymorphic. Therefore, understanding polymorphism is of primary importance when embarking on any drug development/authorization/manufacture/formulation process.

The effort is by no means only theoretical or academic and has important implications both in terms of the drug ultimate efficacy and of the protection of the intellectual property rights associated with the final pharmaceutical product [9].

A number of statistical analyses of the literature have been carried out in an attempt to estimate the extent of polymorphism. A search of the Cambridge Structural Database on the keywords “polymorph”, “form”, “modification”, or “phase” indicates that approximately 4.2% of the ~1,200,000 entries fall into this category. Approximately 25% of the entries are either solvates or hydrates. Other studies based on different selection criteria reveal results falling somewhere between these two extremes [10,11,12,13]. 

It is worthy of note that the “International Conference on Harmonization” [14] includes under the heading of “polymorphs”: “single entity polymorphs; molecular adducts (solvates, hydrates), amorphous forms”. The FDA currently requires that pharmaceutical manufacturers investigate the polymorphism of the active ingredients before clinical tests and that polymorphism is continuously monitored during scale-up and production processes [15]. The European Patent Office also demands the characterization of solid drugs by means of X-ray diffraction to ensure the integrity of the crystal form [16].

## 3. Polymorphism: The Implications. Different Crystals. Different Properties

A truly significant contribution to the understanding of crystal polymorphism in all its numerous facets and industrial implications (from polymorph detection, screening, and assessment to the impact on intellectual property rights in the case of active principles) is due to the work of the late Joel Bernstein and to his intense dissemination efforts [3].

Polymorphs, although possessing exactly the same chemical composition, may differ in a number of properties (see Table 1). The analogy with molecular isomers is strong: if a crystal is seen as a supermolecule then its polymorphic modifications are solid state super-isomers [17]. These isomers may show physico-chemical differences that, in some cases, are as large as to make them behave as practically different species altogether.

**Figure 1:**
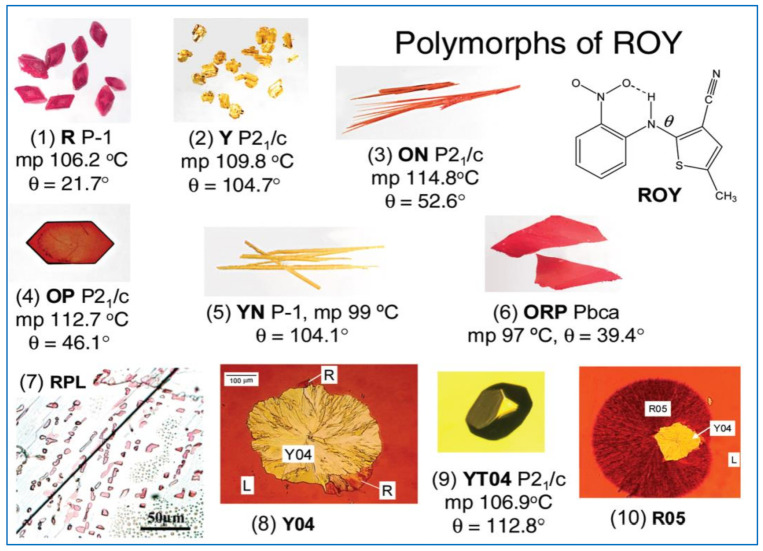
Differences in shape and color between the first ten (out of 13) discovered polymorphs of ROY (ROY = red, orange, yellow polymorphs of 5-methyl-2-[(2-nitrophenyl) amino]-3-thiophene carbonitrile). Reprinted with permission from Ref. [21]. 2010, American Chemical Society.

Even shape and color may differ in a significant manner from form to form with important implications at the manufacturing and processing levels. A striking example is provided by ROY (ROY = red, orange, yellow polymorphs of 5-methyl-2-[(2-nitrophenyl)amino]-3-thiophene carbonitrile), the most polymorphic compound in the Cambridge Structural Database, with its crystal forms differing in color and morphology as shown in Figure 1 [18,19]. The palette of polymorphs of ROY has been recently enriched by the discovery of new ways to search for polymorphs and increase polymorphic diversity, based on crystallization induced by suitably designed mixed-crystal seeds (see also below) [20].

## 4. Polymorphism: The Rationale

Crystal polymorphism is a manifestation of the perpetual thermodynamic–kinetic dualism ruling the physical world. The thermodynamic stability of a polymorph is strictly dependent on pressure and temperature; however, due to kinetic considerations, metastable forms can exist or coexist in the presence of more stable forms [22,23]. 

**Figure 2:**
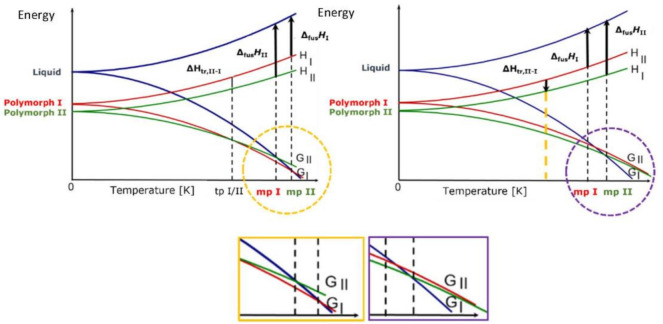
E/T diagrams, with G and H vs. temperature in the enantiotropic (**left**) and monotropic cases (**right**).

For crystals of organic molecules, such as most APIs, the energy difference between different polymorphic forms is usually of the order of few kJ/mol, mainly because of the entropic contribution to the free energy. Polymorphs can be grouped in two major categories depending on whether there is a transition point between two solid phases at a given temperature, i.e., the two phases interconvert via a phase transition, or the two phases do not share a point of identical free energy before melting, i.e., the two phases do not interconvert via a phase transition. In the first case, the two phases are said to be enantiotropically related, while in the second case the two phases are said to be monotropically related (see Figure 2) and will be discussed briefly in the following. 

When polymorphs are enantiotropically related, there is a transition temperature at a temperature below the melting point of the lower melting form. The two crystalline phases are in equilibrium at the transition temperature. The transition temperature is real (Burger–Ramberger Rule 1) and corresponds to ΔG_trans_ = 0, i.e., ΔH = T_trans_ ΔS. Melting is observed only for the polymorph that is stable at a higher temperature (mpI in Figure 2, left).

In the case of a monotropic system, the transition temperature between two crystals is only virtual, since the two G curves only cross in the field of stability of the liquid phase. The two polymorphs have independent melting points (mpII and mpI in the Figure 2, right), and they cannot interconvert in the solid state, as there is no point in the phase diagram where ΔG_trans_ = 0. The transformation can only occur in one direction, from the metastable to the stable form, and cannot be predicted on a thermodynamic ground but may be activated for kinetic reasons. As it will be shown in the following, it is often the case that a thermodynamically metastable crystal form is kinetically favored at the nucleation stage and is crystallized in preference to the thermodynamic form. Therefore, it is often possible to make intentional use of thermodynamically metastable crystal forms to take advantage of very special properties (see below). 

The transformation of a stable to a metastable polymorph in a monotropic system can occur only if it is mediated by a liquid or gas phase, as in fast recrystallization from melt, crystallization from solution, or in vapour digestion processes. Conversion from the metastable to the stable form can be obtained via slurry, or may occur because of changes in pressure, as during a mechanical treatment; it can also be triggered by the presence of impurities. 

In this respect, monotropic systems are the true Sword of Damocles for the pharmaceutical industry, because the interplay of kinetic and thermodynamic factors in a crystallization process is often unpredictable, with consequences that are well known to the practitioners in the area, as it will be discussed below. 

As efficaciously pointed out by Bernstein “it is sometimes difficult to comprehend why and how new polymorphs still emerge (while others disappear) long after crystal-form screens presumably have been completed. [...] The point is that it can never be stated with certainty that the most stable form has been found; at best it can be determined which of the known forms is the most stable. [...] a new (and most often more stable) form can appear at any stage in the history of a compound (or life-cycle of a drug)” [24].

These are the reasons why the “quest for polymorphs” has become a central point in the development of a substance that is administered in the solid form, whether this is a drug, a nutraceutical, a fertilizer, etc. This will be clarified in the following. 

### 4.1. Examples of Polymorphism in Single Component (Unary) Systems

An early textbook example of polymorphism affecting a very commonly used drug is provided by paracetamol. 

**Figure 3:**
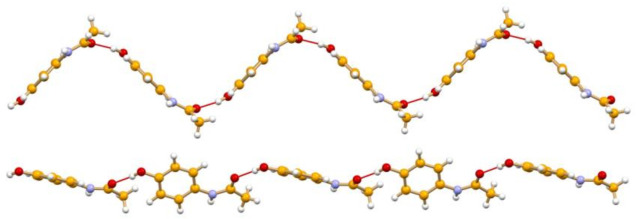
Hydrogen bonded chains in paracetamol Form I (**top**, refcode HXACAN04) and Form II (**bottom**, refcode HXACAN21).

The crystallization of paracetamol from methanol affords a so-called Form I, which although poorly compressible, is the marketed phase. Form I [25] is the thermodynamically stable form at all temperatures, and melts at 440–445 K with a ΔH_fus_ of 26–34 kJ mol^−1^. Crystallization from benzyl alcohol yields the highly compressible Form II [26], which is metastable, and melts in the range 427–433 K with a ΔH_fus_ of 26.4–33.5 kJ mol^−1^. The difference in the hydrogen bonded chains, constituting the basic packing motif in both forms, is shown in Figure 3. A Form III has also been discovered, which can only be stabilized under certain conditions [27]. 

**Figure 4:**
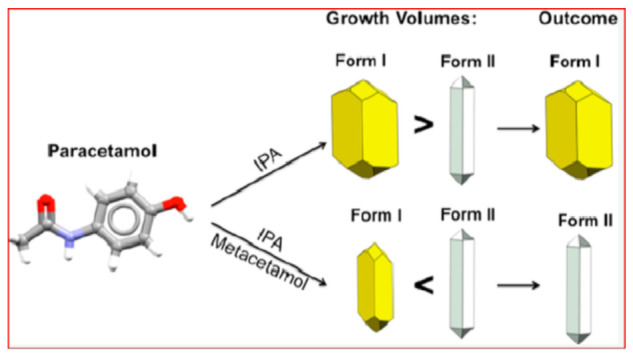
Crystal habits and shapes of the two polymorphs of paracetamol. The outcome of the crystallization can be changed by doping the crystallization with metacetamol. Reprinted with permission from Ref. [28]. 2020, American Chemical Society.

The two forms have different crystal shapes. Moreover, Form II is elusive and hard to crystallize, unless selective impurities are present in solution. Recently, it has been shown that metacetamol, used as an additive, inhibits the growth of Form I and favors the growth of Form II (see Figure 4) [28]. 

Importantly, the two types of paracetamol crystals have also been shown to possess different wettability properties, with consequences on the way the drug is processed and formulated [29].

**Figure 5:**
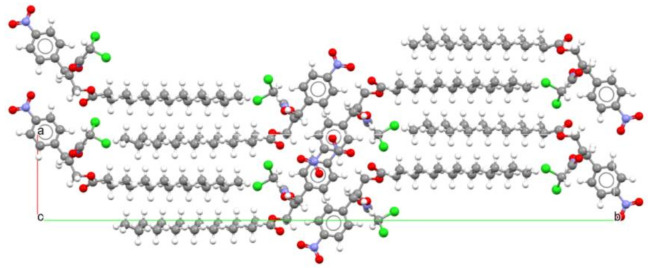
Crystal packing of chloramphenicol palmitate Form B (view down the crystallographic *c*-axis).

An example of the dependence of bioavailability on polymorphic form is provided by the antibiotic chloramphenicol palmitate. Chloramphenicol palmitate exists in three polymorphic forms [30], which recently have been fully characterized thanks to advances in analytical methods. Of these, the so-called forms A and B are monotropically related. Form B is pharmacologically active and is used in suspensions (see Figure 5), while Form A is inactive as an antibiotic. However, Form B is metastable under ambient conditions and, due to its better solubility, in suspension it slowly recrystallizes into form A. Using a thermodynamically metastable modification in the production of tablets, creams, suspensions, and solutions, is sometimes the reason why unwanted changes take place upon storage, caused by transition into the thermodynamically stable modification at ambient conditions. Chloramphenicol palmitate shows that it is possible to make use of thermodynamically unstable crystal forms taking advantage of the considerable kinetic inertness [31].

**Figure 6:**
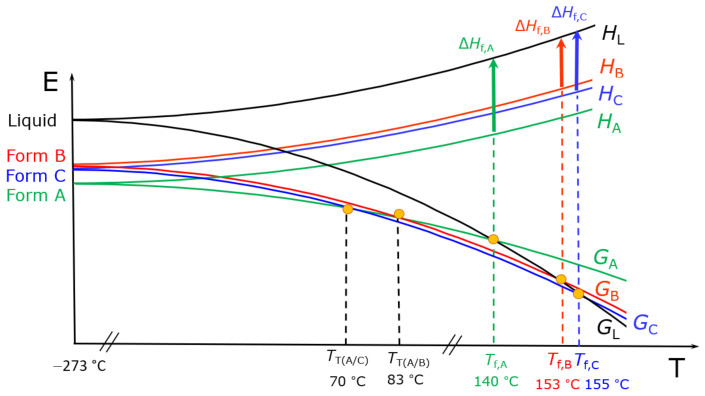
E/T (energy vs. temperature) diagram showing the complex phase relationship between the three unsolvated, non-hygroscopic crystalline forms, designated as form A, form B, and form C of Bitopertin. Figure adapted from [32].

Another example is provided by the drug Bitopertin, which has been shown to possess three unsolvated, non-hygroscopic crystalline forms, designated as form A, form B, and form C [32]. Form A is enantiotropically related to form B, with form A being the thermodynamically stable polymorph below the transition temperature (~83 °C). Form A and form C are also enantiotropically related, with form A being the thermodynamically stable form below the calculated transition temperature (~70 °C). Form B and form C are otherwise monotropically related. The complex relationship between the three phases is shown in Figure 6.

The reader, by now, will have appreciated that the nomenclature of crystal forms is a relevant problem. There is no convention on the naming of polymorphs, with consequences in the understanding of differences and properties of one or another form. This aspect may become particularly relevant when tackling intellectual property issues related to polymorphism.

### 4.2. Conformational Polymorphism

**Figure 7:**
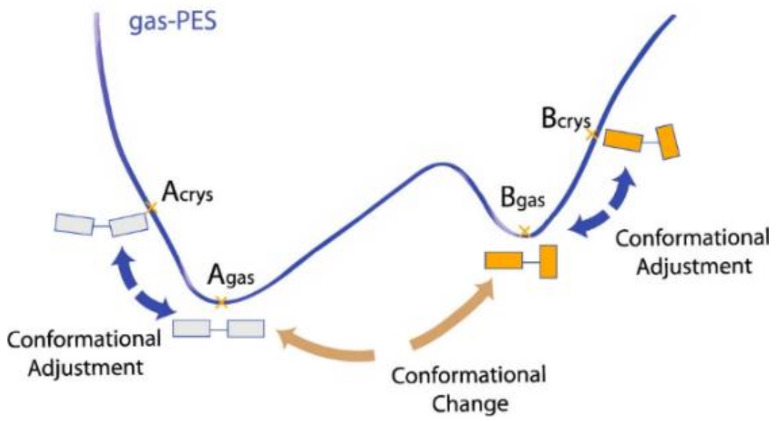
Schematic representation of the concepts of “conformation change” and “conformational adjustment” in a PES (potential energy surface) profile. Reprinted with permission from Ref. [33]. 2014, American Chemical Society.

Conformational polymorphism, viz. polymorphism originated by different molecular conformations in different crystals, is a widespread phenomenon. It has been estimated that ca. 39% of the flexible organic molecules in the CSD exhibit conformational polymorphism [33]. As pointed out by Cruz-Cabeza and Bernstein, however, conformational polymorphism results from “conformational changes”, which should not be confused with “conformational adjustments”. Conformational adjustments occur for any structurally non-rigid molecule in the solid state as a compromise between the optimization of packing energy and the optimization of molecular structure (with respect to the gas-phase unconstrained environment); no energy barrier is involved. Conformational changes, on the contrary, are observed only if an energy barrier separates distinct minima in the intramolecular energy conformational curve, as shown in Figure 7.

**Figure 8:**
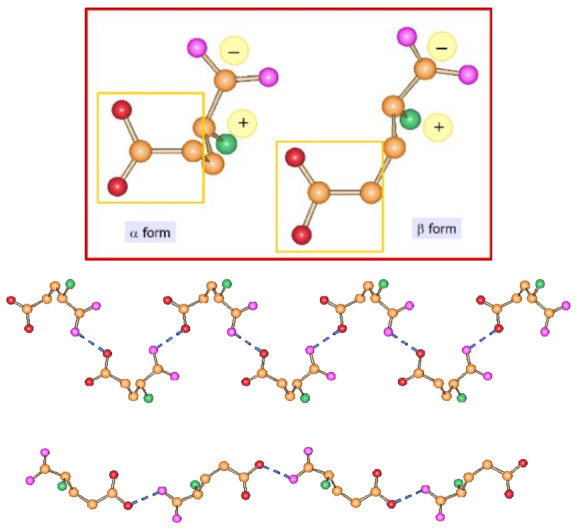
(**Top**) The α and β forms of L-glutamic acid and a comparison of the hydrogen bonded chains in the α (**middle**) and β (**bottom**) polymorphs.

L-glutamic acid is an example of conformational polymorphism. L-glutamic acid, in its zwitterionic form, crystallizes in the two forms α and β, both orthorhombic *P*2_1_2_1_2_1_ (see Figure 8). A detailed thermodynamic investigation of the temperature dependence of the two forms has shown that α-glutamic acid is the preferred form at low temperatures and the β form is most stable at ambient temperatures [34].

### 4.3. Tautomeric Polymorphism

**Figure 9:**
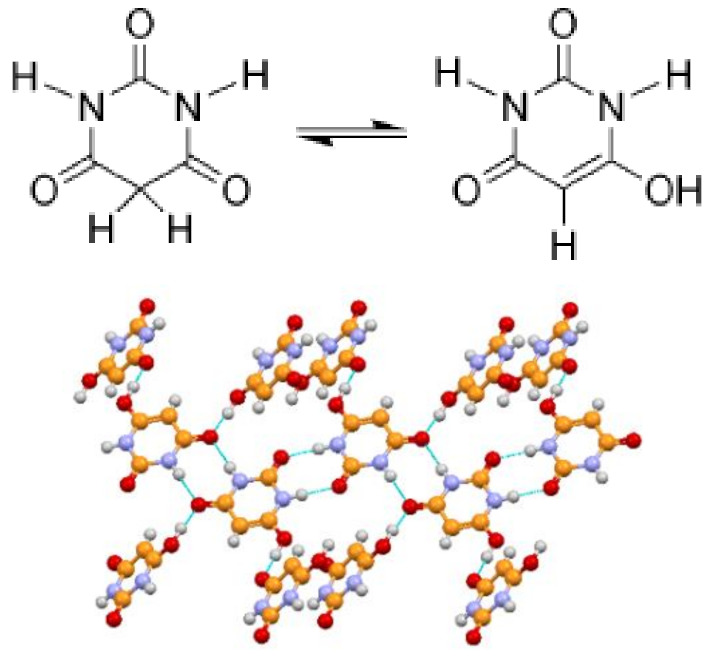
(**Top**): keto/enol tautomerism of barbituric acid; (**bottom**): crystal structure at room temperature of the enol form.

Tautomeric polymorphism may occur due to the crystallization of different tautomers. A textbook example is provided by barbituric acid. The keto form, used in all common representations of this important chemical, is the preferred tautomer in solution; it is also found in two polymorphs and one hydrated form. The stable thermodynamic form, however, as shown by both solid-state NMR and X-ray diffraction [35,36], turned out to be the enol form IV, obtained by the grinding of commercial barbituric acid, or via extremely slow solid-state conversion upon storage at ambient conditions. See Figure 9 for a view of the packing of form IV.

**Figure 10:**
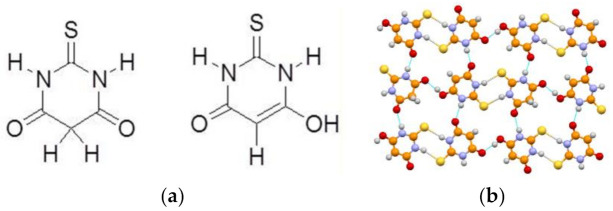
(**a**) Keto/enol tautomerism of thiobarbituric acid and (**b**) the crystal structure of the most stable form at room temperature, showing the presence of both the keto and enol forms [37].

The isostructural compound 2-thiobarbituric acid possesses five polymorphs and one hydrated form. In both the crystalline form II and the hydrate form, the 2-thiobarbituric acid molecules are present in the enol form, whereas only the keto isomer is present in crystalline forms I, III, V, and VI. The stable form IV can also be obtained mechanochemically, as in the case of barbituric acid, and has been shown, by single-crystal X-ray diffraction and 1D and 2D (1H, 13C, and 15N) solid-state NMR spectroscopy, to contain both tautomers in a 50:50 ordered distribution (see Figure 10).

## 5. Polymorphism: The Impact

The examples provided in the previous sections were intended only to give an idea of the spread and complexity of the phenomenon. It was only when some major “polymorphism incidents” severely hit the pharmaceutical industry, however, that the community at large became aware of the “sword of Damocles”.

Undoubtedly, the case of the drug Ritonavir (Norvir^®^) is one of the most striking, also because it had a huge impact on a particularly fragile typology of patients. Norvir^®^ was produced by Abbott and administered for the treatment of HIV. After many years of research, production, and distribution, in 1998, drug production lines begun to show problems related to “undesirable” crystal formation in a series of production batches that failed the dissolution test. An investigation of the reason for the failure showed the unexpected appearance of a new crystalline form of ritonavir that affected the way the drug dissolved, hence its absorption [38]. In spite of all the efforts, Abbott was not able to avoid formation of what turned out to be the thermodynamically more stable, much less soluble, form of ritonavir, form II. Form I and form II are monotropically related with no thermodynamic solid-to-solid transition point at any temperature, hence crystallization of form II could not be predicted.

**Figure 11:**
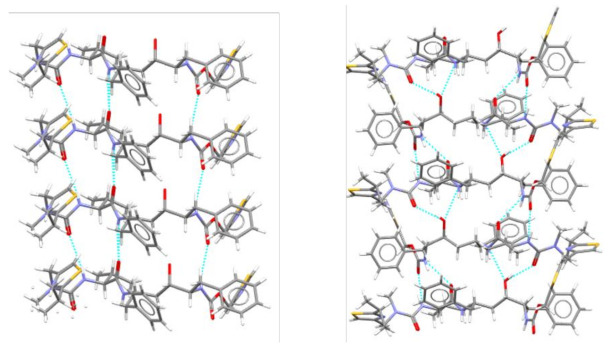
The hydrogen bonding networks in crystals of Ritonavir form I (**left**) and form II (**right**) [38].

In terms of crystal structure, the two forms differ in the relative arrangement of the molecules, which affects the hydrogen bonding pattern, as is shown in Figure 11.

This dramatic incident (the drug was not available for patients till Abbott found an alternative formulation based on soft-gel capsules) was a shock for the pharmaceutical industry and prompted a more thorough investigation of the relative stability of crystal forms. The episode has been thoroughly described in a review by Bučar, Lancaster, and Bernstein [24]. Subsequent investigations led to the discovery of several additional crystalline forms of ritonavir [39,40], all less thermodynamically stable than the “unwanted” Form II.

**Figure 12:**
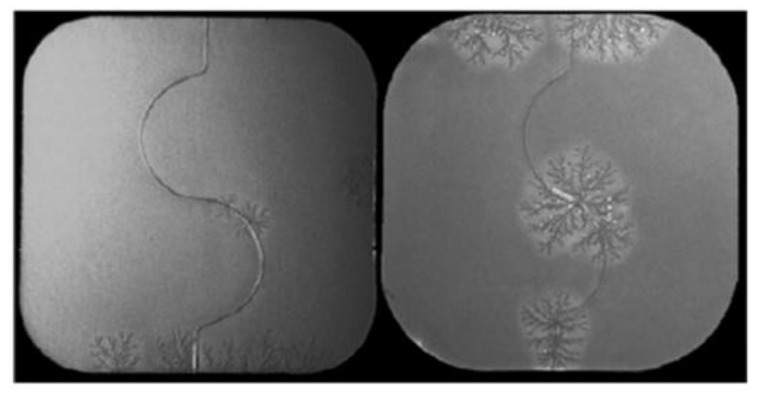
The appearance of the stable crystalline form of Rotigotine on the patches used for administering the drug. Reprinted with permission from Ref. [43]. 2015, Elsevier.

Another important case of unexpected (and unwanted) appearance of a more stable monotropic crystal form of a drug is that of Rotigotine (Neupro^®^), a Parkinson drug produced by UCB and administered to patients as skin patches. In 2008, a new form suddenly appeared, which crystallised in the patches (see Figure 12), reducing the drug efficacy [41]. The product had to be withdrawn from the market with considerable impact on the patients and on the company. The new crystal form was described in a patent filed in November 2008 and granted in July 2012 [42,43].

There are, of course, several other examples of “disappearing (and reappearing) polymorphs”. The interested reader is addressed to the review published by J. Bernstein and others in 2015 [24,44].

### 5.1. Polymorphism: The Reaction of the Scientific Community

Unsurprisingly, the events described above motivated renewed efforts from both the academic and industrial communities. Polymorphism began to be systematically investigated and a number of spinoff companies or new research branches within large pharmaceutical companies were launched. Nonetheless, despite the knowledge of the factors that can cause polymorphs to “appear” (or to disappear), our ability to predict the real occurrence of polymorphism is still embryonic. In most cases, the crystallization of a new crystal form of a substance is still an unexpected event (for example, accidental seeding with impurities may trigger the crystallization of a new, more stable polymorph) rather than the result of a controlled process.

It became clear that the investigation of the crystalline phase(s) of a new API was not simply the characterization of the new pharmaceutical in its solid form, but, rather, the beginning of a long journey in the quest for crystalline materials with controllable properties. This journey required specific skill and training and also access to a variety of solid-state methods and techniques to be used in combination. The objective is that of minimizing, if not eliminating, the chances of an unexpected appearance of unknown new crystal forms of a drug at later stages of its development, or even when the drug is already on the market.

It is now clear that it is not only necessary to explore as thoroughly as possible the “crystal space domain” of the molecule of interest, but also to be able to follow the production, storage, and distribution of the product to guarantee persistence of the solid form, hence of the selected properties. 

**Figure 13:**
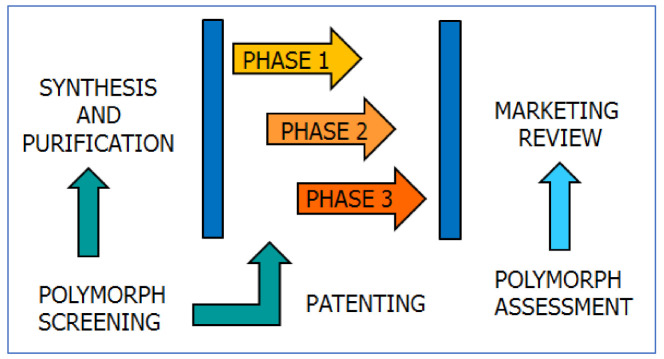
A flow-chart showing how polymorph screening ought to be associated with the API selection, before entering trial stage, and the quality control process, once the API is on the market.

Polymorph assessment has indeed become part of the system of quality control in the pharmaceutical industry [4,5,6]. It is necessary to make sure that the scaling-up from laboratory preparation to industrial production does not introduce variations in crystal forms. Polymorph assessment also guarantees that the product conforms to the guidelines of the appropriate regulatory agencies and does not infringe on the intellectual property protection that may cover other crystal forms. The schematic diagram in Figure 13 shows how polymorph screening can lead to relevant patenting in the process of drug development, while continuous polymorph assessment will be required once the drug is on the market.

Both initial polymorph screening and continuous crystal form assessment require the combined use of several solid-state techniques, among them (not exclusively or in any preferential order): microscopy and hot stage microscopy (HSM), differential scanning calorimetry (DSC), thermogravimetric analysis (TGA), infrared and Raman spectroscopy (IR and Raman), single crystal/powder X-ray diffraction (SCXRD, PXRD), and solid state nuclear magnetic resonance spectroscopy (SSNMR) [45]. The discussion of these techniques and of the pros and cons and pitfalls is well beyond the scope of this review. 

Clearly, in the pharmaceutical field, the screening of crystal forms is motivated by safety and commercial necessities and is relevant in terms of patenting and in general of intellectual property protection. Interestingly, this has had repercussion also for fundamental science. Litigations over polymorphs and hydrates, in addition to the polymorphism “incidents” mentioned above, have fueled research in solid-state chemistry, and oriented the experience and competence of many academic research groups worldwide. The birth of many spinoff companies, responding to the demand of industrial outsourcing of accurate solid-state investigation, ought also to be mentioned.

### 5.2. Crystal Structure Prediction

On closing this section, it is also important to mention the increasing importance that is being acquired by computational crystal structure prediction (CSP). The term “crystal structure prediction” (CSP) comprises computational methods to explore the thermodynamic domain of molecular crystals. CSP aims to find the most thermodynamically stable crystal structure of a given molecule by evaluating the crystal energy landscape. In this respect, crystal structure prediction is complementary to experimental screening and provides information on the existence and relative energies of polymorphs [46,47]. 

As we have seen, if polymorphs are enantiotropically related, the stable structure depends on the temperature, i.e., polymorphs may interconvert. If the polymorphs are monotropically related, thermodynamically metastable polymorphs may be kinetically inert and persist indefinitely because of the difficulty in transforming into the stable form. The cases discussed above of ritonavir and rotigotine are emblematic examples of the consequences of the late appearance of more stable crystal forms. It would thus be of paramount importance in the development of a new drug, or of any new material where the solid form properties are essential for its utilization, to reach a good level of confidence on the relative thermodynamic stability of the crystal phase under examination. Even more so it would be of great value to be able to design ab initio the most stable crystal structure of a given molecule.

Because of polymorphism, computed crystal energy landscapes invariably contain several crystal structures separated by small differences in energy. Hence, the main use of CSP is to explore the range of packings and relative energies of the thermodynamically feasible crystal structures. A successful CSP would always generate the most stable crystal structure; the same structure, however, would invariably be obtained experimentally only if the crystallisation process were completely under thermodynamic control. 

A detailed discussion of CSP is beyond the scope of this review. The interested reader is addressed to a more specialized literature [46,48]. It is worth mentioning, however, that crystal structure prediction methods are periodically assessed via the Blind Tests organized by the Cambridge Crystallographic Data Centre [49]. The Blind Test is based on a collection of unpublished crystal structures, which are sent out in the form of chemical diagrams to those developing CSP methods, with the challenge to submit predictions of the crystal structure by a given deadline.

The sixth blind test of organic crystal structure prediction was held in 2016 over five target systems constituted of a small nearly rigid molecule, a polymorphic drug candidate, a chloride salt hydrate, a co-crystal, and a bulky flexible molecule (see Table 2). The challenge saw the participation of 25 teams [50].

All targets, apart from a single potentially disordered polymorph of the drug candidate, were predicted by at least one participating group, each group being allowed to propose up to 100 predicted structures ranked in order of likelihood [50]. 

Overall, the results of the 2016 Blind Test demonstrates the increased maturity of CSP methods and shows how CSP calculations can guide and complement our understanding and experimental studies of organic solid forms.

The seventh crystal structure prediction blind test, organized by the CCDC, was launched in October 2020 and registration closed on 14 June 2022: results will be presented at the end of September 2022 [51].

The fact that the most stable structure in CSP is not always observed reflects the limitations of the thermodynamic modelling of crystallization. Kinetic effects on the nucleation and growth of less stable crystal forms are not taken in account. Moreover, a crystallization in the “real world” (in vitro, not in silico) implies the use of solvents and compounds with a purity profile determined by the detection methods and also of hardware (glassware and instruments) with the possible release of microparticles, all implying the possibility of unintentional seeding and other physical effects that may favor the nucleation of less thermodynamically stable forms.

However, CSP is progressing rapidly. The increasing success of the Blind Tests indicate that the future will show a wider utilization of CSP to guide the experimental work in the quest for “missing” polymorphs [48].

## 6. Solvates and, Especially, Hydrates

An API can form polymorphs, i.e., different crystals of the same chemical entity; however, an API can also form different crystal structures with solvent molecules, i.e., solvates [52]. Crystalline solvates may present different stoichiometries, i.e., mono-, di-, tri-solvates, etc. or they can be non-stoichiometric. In this latter case, they adsorb/release a variable number of solvent molecules depending on the temperature, relative humidity (in the case of hydrates), or other physical conditions. Solvate formation is also largely unpredictable. Solvates are sometimes called “pseudo-polymorphs”, but this practice ought to be discouraged, since solvates have a different chemical composition from the pure API. Moreover, crystalline solvates can show polymorphism, i.e., the same compositions, the same API/solvent stoichiometric ratio, but different crystal structures. 

When crystalline materials are being used for living beings, the permitted solvents are often restricted to water and very few bio-compatible (GRAS: generally regarded as safe) solvents [53]. Clearly, in the case of an API, hydrates are not only common but are also amply manageable and fully acceptable in formulation.

For this reason, we will hereafter focus on hydrates, with the understanding that solvates and hydrates share much in terms of the methods of characterization and analysis. In fact, precipitation from a solution either by solvent evaporation or by a temperature gradient is the most common way to obtain crystals. In these conditions, the formation of a solvate is an unsurprising event. In the case of hydrates, it is also very common that water is taken up from glassware, reactants, and solvents. The situation is further complicated by the ubiquity of water [54]. The formation of hydrates, though not certain, is indeed very common [52,55].

A statistical analysis based on the crystal structures deposited in the CSD [56] (until 2016) showed that approximately 7–8% of the organic crystal structures are in the form of hydrates, whereas only 1.4% form single entity polymorphs, as listed in Table 3.

**Figure 14:**
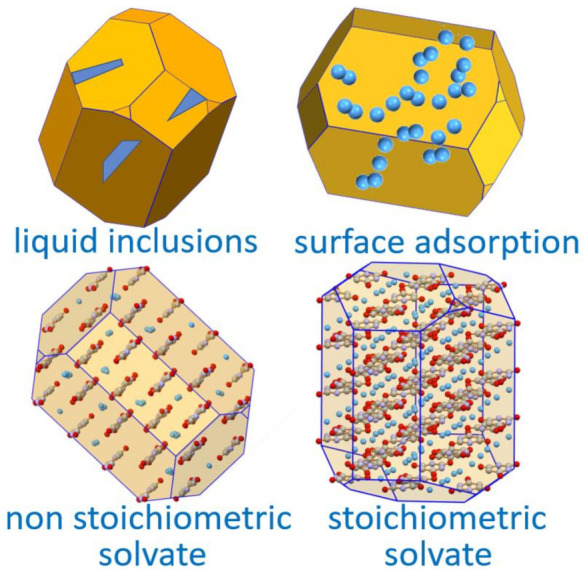
A representation of the types of association of a solvent with a crystalline solid.

The association of water with a crystalline material can take different forms [52]. Water may form stoichiometric hydrates, whereby water molecules are linked, generally via a hydrogen bond and/or via coordination of the oxygen atoms to other atoms in the crystal, or may be absorbed in disordered regions or cracks and cavities within the crystal mosaic or adsorbed on the crystal surface (Figure 14). This is an important notion to keep in mind when evaluating the amount of water present in a crystalline material, especially when the extent of hydration is a relevant aspect for the utilization of the crystalline material, as in the formulation of pharmaceuticals.

### Stoichiometric versus Non-Stoichiometric Hydrates

As mentioned above, crystalline hydrates can be non-stoichiometric, i.e., may absorb a variable number of water molecules in the crystal structure, depending on the environmental conditions such as humidity and temperature. The most relevant structural difference between a stoichiometric and a non-stoichiometric hydrate is that, in the former case, hydrate and anhydrate usually possess different crystal structures, whereas in the non-stoichiometric case the features of the crystal structure are retained (almost) unaltered upon absorption and release various amounts of water. This is made possible by the presence, in the structure of non-stoichiometric hydrates, of channels or cavities that allow the entrance, transit, and exit of water molecules without significantly affecting the overall packing.

The stoichiometric versus non-stoichiometric nature of the hydrate can be assessed by dynamic vapor sorption (DVS), a technique that allows us to establish whether water uptake is continuous as the humidity increases or passes through an abrupt change, which accompanies the crystal structure transformation from anhydrate to hydrate. In the stoichiometric case, anhydrous and hydrated crystals show distinctly different physicochemical properties (e.g., aqueous solubility) and hysteresis between hydration and dehydration. If the structures are investigated by diffraction, they will very likely show different unit cells and a different organization of the molecules. In the non-stoichiometric case, on the contrary, the difference in the physico-chemical properties will be small and the solid phases will show a highly variable composition in terms of water content, but a very small variation in cell parameters and overall structure organization. 

Ampicillin and theophylline are good examples of the dependence of hydrate formation on water activity in organic solvents for the compounds.

**Figure 15:**
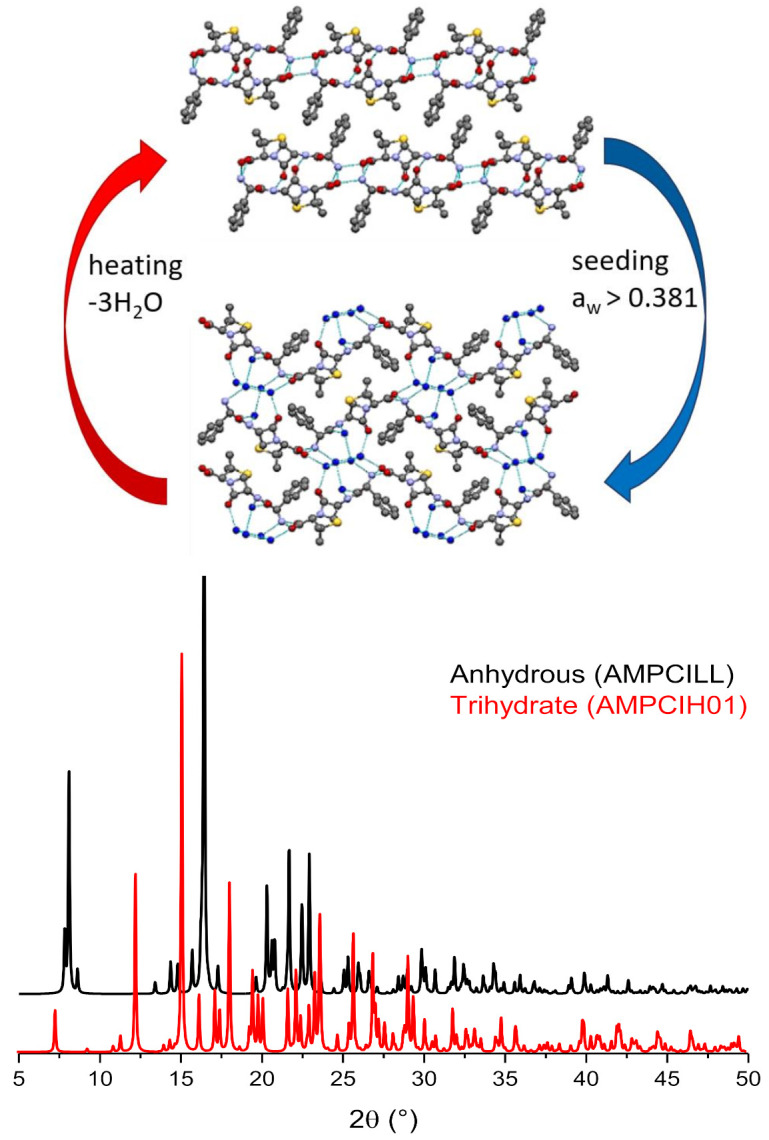
(**Top**): packing comparison and interconversion process between anhydrous and trihydrate ampicillin (refcodes AMCILL and AMPCIH01, respectively). (**Bottom**): comparison between the powder diffractograms calculated on the basis of the single-crystal data, showing the difference between the two patterns.

Anhydrous crystalline ampicillin is kinetically stable for several days in MeOH/H_2_O mixtures over the whole range of water activity. Even though at ambient conditions the crystalline trihydrate is more thermodynamically stable and less soluble than the anhydrate, conversion to the hydrated form occurs only with the addition of seeds at a water activity >0.381 [57] (see Figure 15).

**Figure 16:**
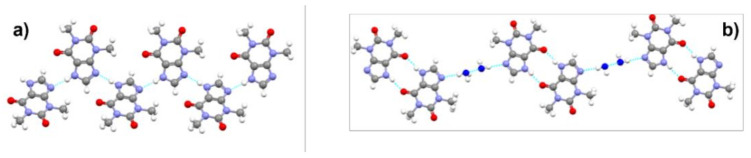

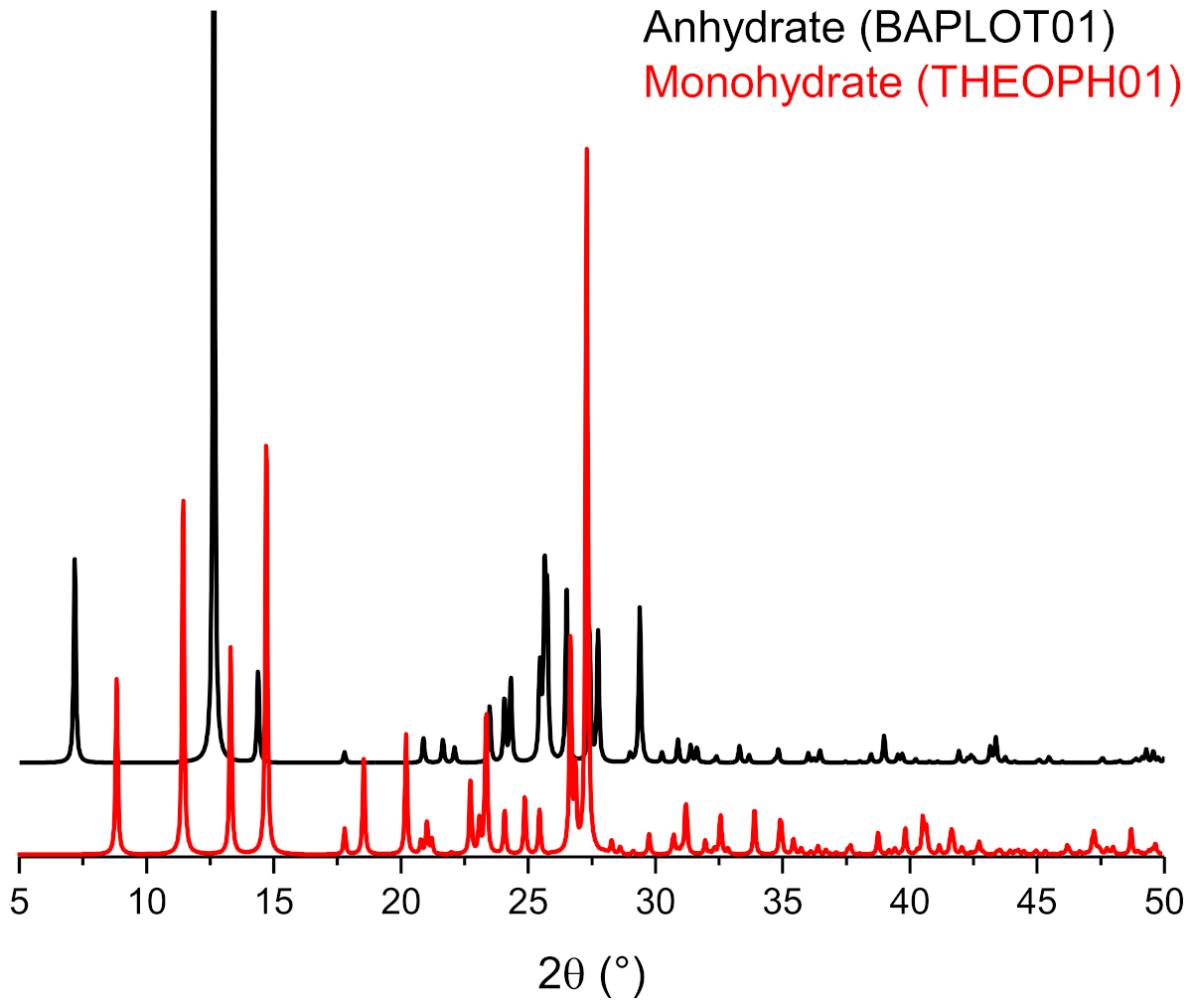
(**Top**): The structures of the anhydrate ((**a**), refcode BAPLOT01) and of the monohydrate ((**b**), refcode THEOPH01) crystals of theophylline. The anhydrate is obtained from a mixture of an organic solvent and water at low water activity. (**Bottom**): comparison of the the powder diffractograms calculated on the basis of the single-crystal data, showing the difference between the two patterns.

Theophylline forms an anhydrate and a monohydrate that interconvert in organic solvents, depending on the water activity. At a low water activity (<0.25) the anhydrate is the only species present, whereas at a higher water activity the monohydrate is the most stable form [57,58] (see Figure 16).

Although hydrate formation can sometimes be reversed by the drying process, the dehydration of hydrates can lead to the formation of amorphous material or crystal defects that can have potential deleterious effects on physical and chemical stability [59,60]. Hydrates are generally expected to be thermodynamically more stable, hence less soluble and slower to dissolve than anhydrates above the critical water activity for hydrate formation. Hence, dehydrated hydrates tend to be metastable with an easy uptake of water (or other solvents).

An interesting notion, albeit slightly counterintuitive, is that most organic hydrates are less soluble in water than the corresponding anhydrous compounds at the same temperature, as shown by the solubility data compared in Table 4. As an example, the solubilities of anhydrous and hydrated crystals of caffeine are 49.7 and 21.8 mg/mL, those of carbamazepine 0.424 and 0.139 mg/mL, and those of sulfaguanidine 1.38 and 1.07 mg/mL, respectively [59]. The reason is that in hydrates, some of the water–molecule interactions, mainly taking place via hydrogen bonds, are already satisfied in the crystals, thus decreasing the solvation energy contributions to dissolution.

Hydrates are also patentable because they often meet the required non-obviousness and innovativeness criteria. In terms of novelty, anhydrates and hydrates, often because of their difference in solubility (see above), have properties that are distinct from those of polymorphs and other solvates of the same API. One should keep in mind that, even when crystallization takes place from water, it is difficult if not impossible to predict whether a stable hydrate might precipitate out, let alone its physico-chemical properties. 

A cautionary word is in order: the water content, as determined from diffraction data, is averaged over all unit cells forming the crystals used for that specific experiment. In the case of polycrystalline material, however, adsorbed water might be present, and this may impact on the water content established by analytical methods (e.g., Karl Fischer titration) [55]. Care should thus be taken when comparing information coming from diffraction and analytical methods, especially in the cases of non-stochiometric hydrates (see below).

**Figure 17:**
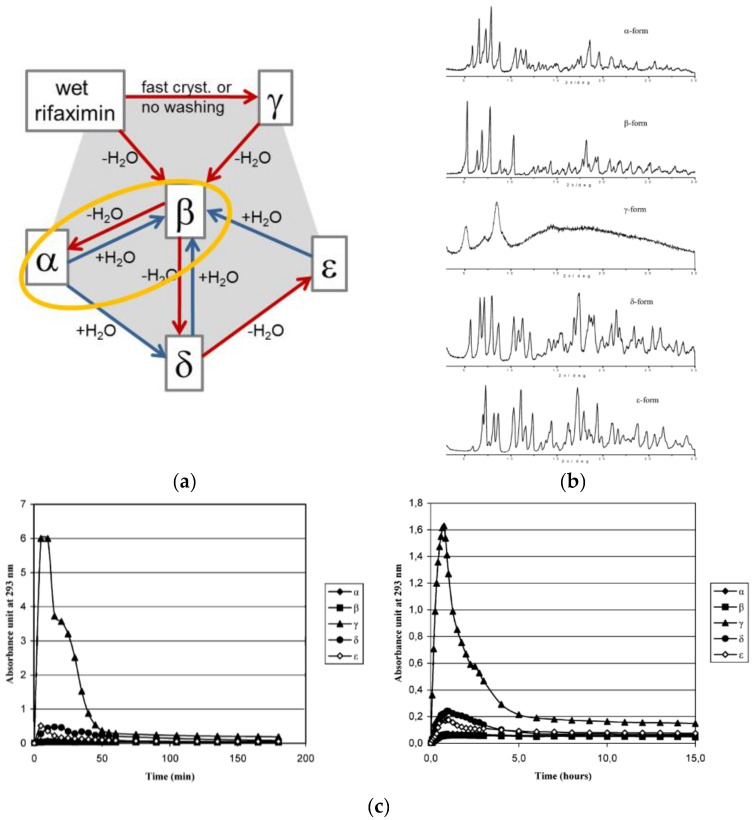
(**a**) The intricate hydration/dehydration phase relationship between the various forms of Rifaximin, (**b**) the powder X-ray diffraction patterns five forms of Rifaximin, (**c**) dissolution rates of Rifaximin hydrates at 250 (**left**) and 100 (**right**) rpm of mixing rate. Time vs. absorbance at 293 nm. Reproduced with permission from [63,64].

A good example of a commercial drug with important properties depending on the degree of hydration is provided by Rifaximin. Rifaximin, (4-deoxy-4′-methylpyrido[1′,2′-1,2]imidazo-[5,4-c] rifamycin SV) is a synthetic antibiotic; its mechanism of action relies on the inhibition of bacterial RNA synthesis by binding the β-subunit of the deoxyribonucleic acid (DNA)-dependent ribonucleic acid (RNA) polymerase [61,62]. Several distinct crystalline forms of Rifaximin have been reported [63,64]. They are basically non-stoichiometric hydrates that interconvert depending on the amount of water and on the way dehydration/hydration is carried out. What is important is that they exhibit remarkable differences in water solubility ranging from 2 mg/L up to more than 40 mg/L, which deeply affects the Rifaximin bioavailability. It is thus of paramount importance to be able to clearly identify the different hydrates, and this can be done via powder X-ray diffraction (see Figure 17). 

**Figure 18:**
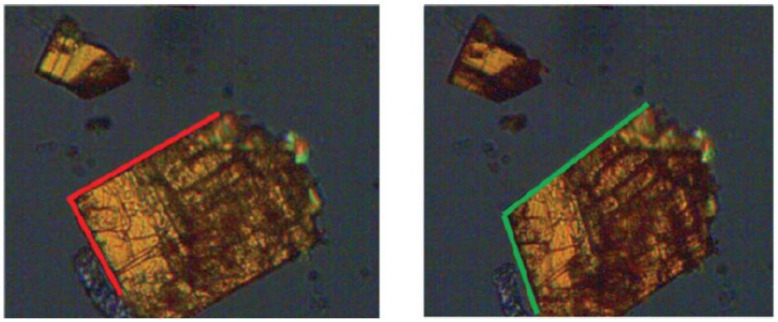
The single crystal to single crystal form β → form α transformation. The red and green lines evidence the change in the monoclinic β-angle from 91° in Rifaximin form α to 110° in Rifaximin form β. Reprinted with permission from Ref. [63]. 2019, Royal Society of Chemistry.

Form α, a form with a low water content, can only be obtained by the dehydration of form β. Figure 18 shows that transition β → α-form on heating on a hot stage microscope a single crystal of the β-form. It is noteworthy how the β-form at 25 °C (monoclinic β angle, shown in red, ca. 91°) transforms at 50 °C into the α-form with the monoclinic β angle changing to ca. 110°.

**Figure 19:**
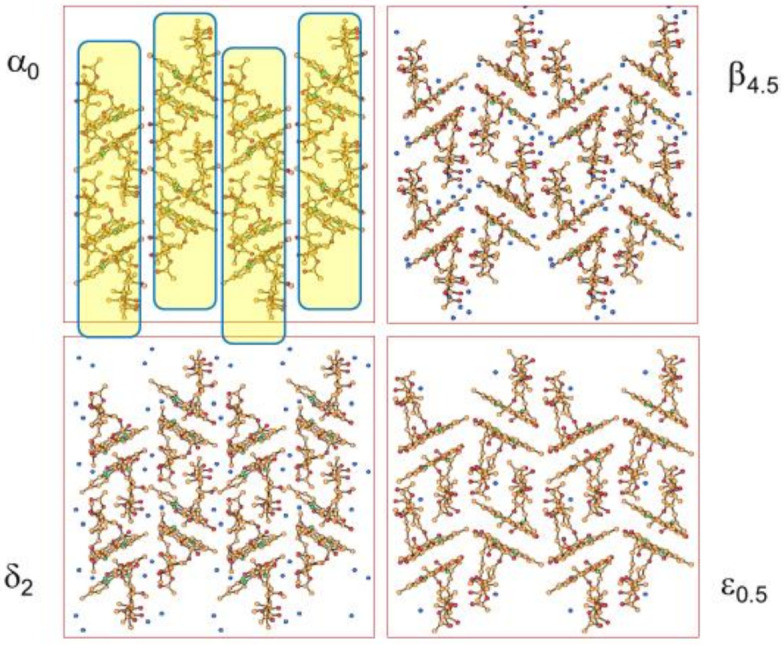
A comparison of molecular packings in four hydrate forms of the antibiotic Rifaximin. Reproduced with permission from [63].

The relationship between the different crystal phases can be better appreciated by comparing the structures determined by single-crystal X-ray diffraction (see Figure 19). The packing in all forms of Rifaximin can be seen as obtained by a juxtaposition of “molecular rods” formed by pairs of Rifaximin molecules. In addition to a change in the relative orientation of Rifaximin molecules within a dimeric pair, forms α, β, δ, and ε present a different relative arrangement of the molecular rods, which seem to “slide” on passing from one form to the other.

Crystal forms of Rifaximin solvates with organic solvents are also known in the patent literature [65,66]. 

**Figure 20:**
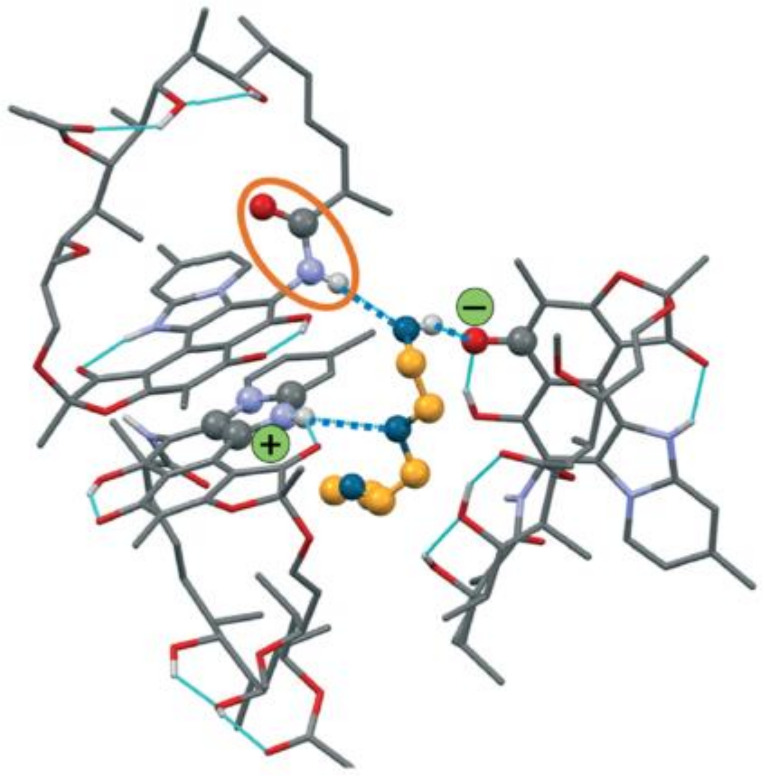
The solid-state structure of Rifaximin τ, showing the interaction of Rifaximin with transcutol^®^ [67,68]. Reprinted with permission from Ref. [67]. 2019, Royal Society of Chemistry.

An interesting example of how dissolution rate profiles may be altered, and hygroscopic behavior improved by the formation of solvates is that of the recently reported and patented Rifaximin τ, a transcutol^®^ (transcutol^®^ IUPAC name 2-(2-ethoxyethoxy)-ethanol) solvate crystal shown in Figure 20.

**Figure 21:**
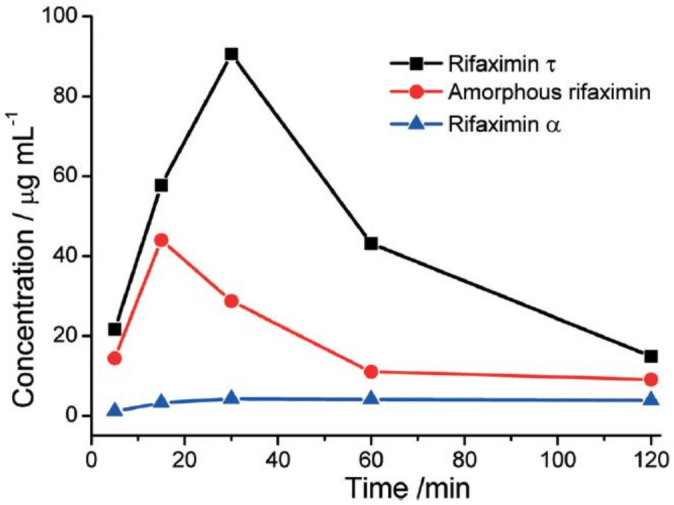
Comparison of dissolution rates for Rifaximin τ, amorphous Rifaximin, and Rifaximin α at neutral pH. Reprinted with permission from Ref. [67]. 2019, Royal Society of Chemistry.

The comparison of the dissolution rate profiles in Figure 21 clearly shows how the dissolution rate of Rifaximin τ is higher than the one observed for amorphous Rifaximin.

## 7. Molecular Co-Crystals

After discussing polymorphs and hydrates, we will now review the topic of co-crystals. Undoubtedly co-crystals have become one of the major attractions for all those interested in altering the physico-chemical properties of APIs or in finding new ones. This is because the association in the solid state of two or more chemically distinct entities, each forming stable solid phases at ambient conditions, is proving to be one of the most fruitful ways to modify solid state as well as biological properties of the crystals of active molecules. This can be applied to pharmaceuticals already in use and/or to access different pharmacological properties by combining different drugs in one crystalline material (co-drugs).

**Figure 22:**
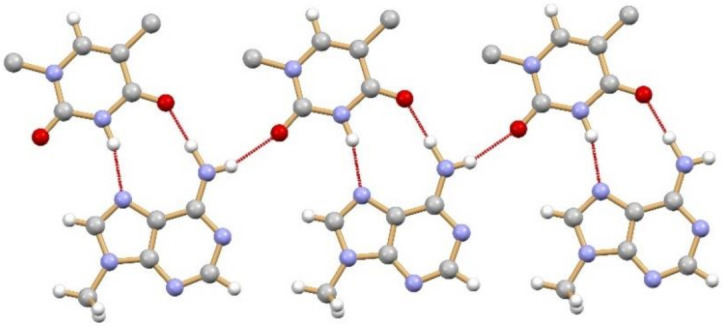
Hydrogen bonding pattern in the 1:1 co-crystal of 1-methyl adenine and 1-methyl thymine, first reported in 1963 by Hoogsteen (CSD refcode MTHMAD).

The term co-crystal was used for the first time in 1963 by Hoogsteen when he reported the structure of an adduct between 1-methyl adenine and 1-methyl thymine (CSD refcode MTHMAD, see Figure 22) [69].

The first hint to the possibility of using co-crystallization as an instrument to modify solid state properties can probably be found in a 1991 paper by Margaret Etter [70], where she discussed “ways to prepare organic crystals and to use co-crystallization to probe the forces involved in aggregation phenomena” and “how molecular aggregation can impart unexpected new properties to organic compounds”.

As a matter of fact, the very definition of a co-crystal is not straightforward, and has been addressed in slightly different ways by various authors. In 2003, J. Dunitz defined a co-crystal as “a crystal containing two or more components together” [71]. In 2004, M. Zaworotko and O. Almarsson provided a definition of a pharmaceutical co-crystal as “a stoichiometric multiple component crystal in which at least one component is molecular and a solid at room temperature (the co-crystal former) and forms a supramolecular synthon with a molecular or ionic API” [72]. In 2005, C. Aakeröy and D. J. Salmon preferred “compounds constructed from neutral molecular species [...] that are solids at ambient conditions [...] and [...] present in definite stoichiometric amounts” [73].

In the context of this review, the definition of a co-crystal as “a multicomponent crystal formed by two or more compounds that are solid at RT and that interact via non-covalent bonding” has been adopted. A corollary of this definition is that a co-crystal is not a solvate (solvent molecules are not solid at RT) and is not a salt (ions do not have separate identities) but could be the association of a neutral molecule with a coordination compound or with an organic or inorganic salt. In this latter case, the definition of ionic co-crystals is adopted. 

Indeed, co-crystals may offer new ways to design or to alter the properties of solid active ingredients including the thermal stability, the shelf life, the solubility, the dissolution rate, the compressibility, etc., by linking the co-crystal former with a suitable ancillary molecule, a co-former. Obviously, these ought to be GRAS molecules for pharmaceuticals [53]. If the co-former happens to be another API, the co-crystal is a co-drug, with all the implications for the regulatory process and authorizations. It is important to appreciate that the differences in physico-chemical properties between a co-crystal and the parent single-molecule crystal are usually larger than those between polymorphs and often also than those between the active ingredient and its solvates/hydrates.

**Figure 23:**
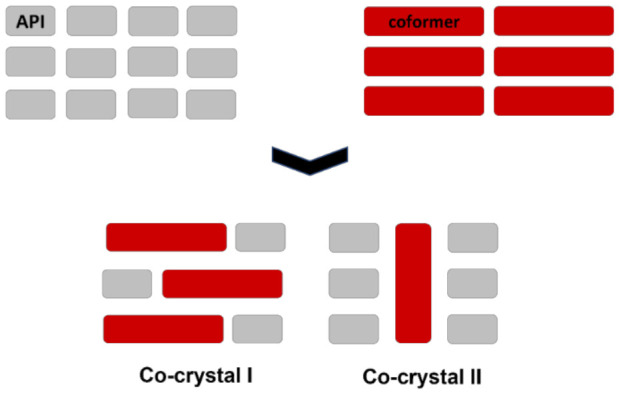
A schematic representation of two polymorphs of a co-crystal of the same API and a conformer.

Co-crystals may also be polymorphic. Figure 23 shows a schematic representation of two polymorphs of a co-crystal of an API and a conformer.

### 7.1. Co-Crystals in Pharmaceutics

For the reasons outlined above, it should be clear why the topic of pharmaceutical co-crystals is being extensively investigated. The subject has been addressed in a number of books, publications, and reviews. Good entry points are the books cited above [6,7], while reviews on the subject matter, with a focus on pharmaceutical co-crystals, were written by Zaworotko et al. in 2016 [74] and, very recently, by Nangia et al. [75]. The occurrence of polymorphism in multicomponent systems, including co-crystals, has also been reviewed recently [76].

**Figure 24:**
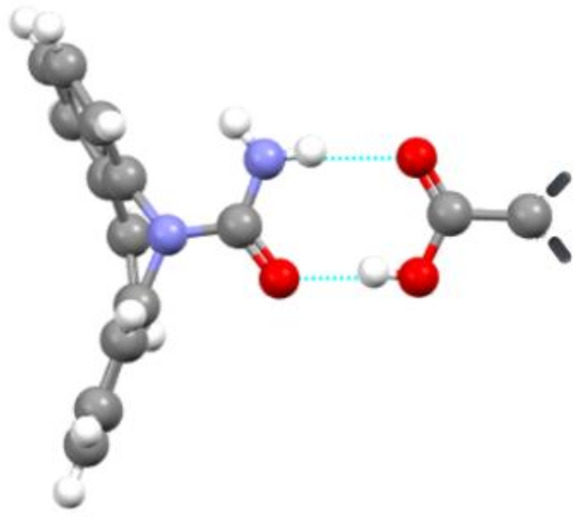
The acid–amide synthon frequently occurring in the co-crystals of carbamazepine with carboxylic acids.

As an example, it is worth citing the thorough exploration of the co-crystal domain of carbamazepine with a series of pharmaceutically acceptable carboxylic acids, as reported by Childs et al. [77]. The authors not only explored a large number of co-formers, but also tested and compared four different screening techniques to form co-crystals. Out of this screening, 27 co-crystals with 18 carboxylic acids were generated and characterized both by XRD (see Figure 24).

Within the broad family of co-crystals, ionic co-crystals deserve a special mention, as they contain ionized components together with an API or an API precursor [78]. The ionic co-former may be either a salt of an ionizable molecule (carboxylic acid, amine, etc.), or a metal salt, e.g., LiCl. The stability of the ionic co-crystals depends on the interactions established by the organic moiety with cations and anions; usually oxygen or nitrogen atoms donate electrons towards the cation, while hydrogen bonds are formed between hydrogen donor groups on the organic moiety and the anions. Therefore, they resemble the interactions that a solvent molecule might establish with ions in solution.

An example of an ionic co-drug co-crystal co-drug is provided by the co-crystals [79] obtained by reacting LiCl (a drug used as a mood stabilizer in patients affected by bipolar disorder) together with piracetam, a cognition enhancing medicine (Nootropil^®^). The preparation of these materials can be easily attained by mechanical mixing of the API (in this case piracetam) with LiCl, but also with other salts such as LiBr as well as with other bio-compatible inorganic salts [80,81]. 

It is worth mentioning, on passing, that ionic co-crystals have also been used in the food sector long before the subject of co-crystals gained popularity. The preparation of compounds based on the association of sugars with inorganic salts dates back more than a century. Since combining NaCl with carbohydrates allows for the introduction of a combined source of sodium and calories, the idea of ionic co-crystals with sugars is rather interesting from a nutraceutical point of view. A number of carbohydrates have been found to form stable co-crystals with NaCl, in particular pentoses (e.g., ribose, arabinose, and xylose), hexoses (e.g., glucose, fructose, galactose, and mannose), as well as disaccharides (e.g., sucrose, lactose, and trehalose). The subject of ionic co-crystals with carbohydrates has been reviewed by Oertling [82].

### 7.2. Co-Crystals Properties

Compared to polymorphs, solvates and co-crystals are more likely to induce significant changes in the solid-state properties of the active ingredient. Clearly, whether additional pharmaceutical and clinical tests might be required will depend on the nature of the coformer, especially if it does not belong to the molecules admitted as GRAS.

In many pharmaceutical applications, the key issues are often, but not exclusively, those related to the solubility and/or dissolution rate of the active molecules of interest. This aspect is also of interest from a patenting point of view, because it may allow an extension of the intellectual property protection of an active ingredient.

A number of authors have explored the preparation of pharmaceutical co-crystals to attain better solubility or better dissolution rates [83,84]. The reader may refer to Good and Rodriguez-Hornedo (2010) [85] for an example of evaluation of the factors controlling and affecting co-crystal solubility. Several studies have been reported on the intrinsic dissolution rates of co-crystals. For instance, in the case of the co-crystals of poorly soluble 2-[4-(4-chloro-2-fluorophenoxy)phenyl]pyrimidine-4-carboxamide with glutaric acid, a dissolution rate 18 times higher than that of the pure API was observed [86].

**Figure 25:**
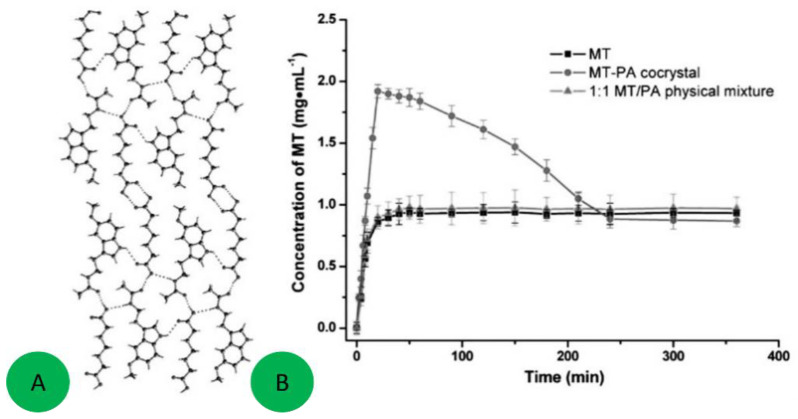
Packing diagram of the melatonine/pimelic acid co-crystal showing the hydrogen bonding (**A**). Powder dissolution profiles of melatonine, melatonine-pimelic acid co-crystal, and of a 1:1 physical mixture in PBS (pH 6.8) at 37 °C (**B**). Adapted with permission from Ref. [87]. 2015, Royal Society of Chemistry.

**Figure 26:**
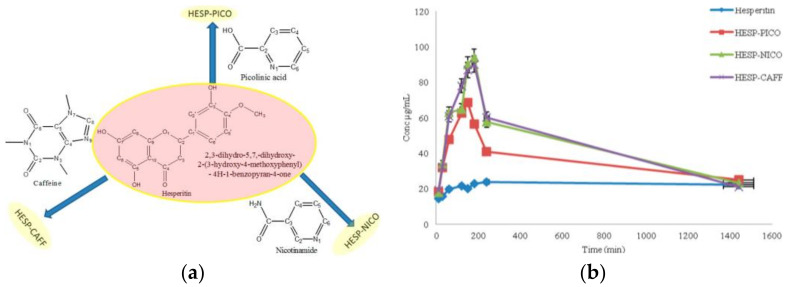
(**a**) The effect of co-crystallization on the dissolution rate for co-crystals of hesperetin with picolinic acid, nicotinamide, and caffeine. (**b**) Equilibrium solubility (24 h) of hesperetin, HESP-PICO, HESP-NICO, and HESP-CAFF. Reprinted with permission from Ref. [89]. 2017, American Chemical Society.

The co-crystal of melatonin with pimelic acid is another example (Figure 25) [87]. The oral bioavailability of melatonin in humans is limited and efforts to improve the dissolution rate and solubility are ongoing. Co-crystal formation occurs when a 1:1 molten mixture of melatonin and pimelic acid is allowed to crystallize in the temperature range 50–70 °C. The co-crystal displays a significantly higher apparent solubility and acceptable stability as compared to the original melatonin. Figure 26 also shows that the melatonin concentration in the end drops back to the equilibrium solubility level of the pure drug, but the 3 h of increased solubility can be crucial for obtaining a higher bioavailability. 

Dissolution enhancement can also be found in a number of different crystal forms of niclosamide, an active ingredient belonging to the salicylamide class [88], obtained by solvent-free synthesis and characterized by diffraction, thermal methods, and solid-state NMR spectroscopy (1H, 15N, 13C CPMAS). Since niclosamide has a very poor aqueous solubility in water, it has proven extremely important to increase its dissolution rate via the formation of ionic co-crystals. 

The effect of co-crystallization on the dissolution rate has been studied in the case of the co-crystals of hesperetin with picolinic acid, nicotinamide, and caffeine, shown in Figure 26a. The dissolution rate experiments in Figure 26b show the parachute effect accompanying dissolution of the three co-crystals, with solubility reaching a maximum concentration in a short time then dropping down, first abruptly, then slowly but steadily, approaching the levels shown by pure hesperetin. This change in solubility is explained by the breakdown of co-crystals to the starting molecules on extended exposure to an aqueous medium, as is confirmed by FT-IR analyses of residues.

### 7.3. Chiral Resolution via Co-Crystallization

Co-crystallization is a viable route to the resolution of racemic mixtures. There are, basically, two main strategies for chiral resolution via co-crystal formation. The first approach is based on the preparation of a chiral host compound with cavities capable of forming inclusion complexes with only one of the enantiomers of the guest molecule. In the second approach, an enantiopure co-former is co-crystallized with a target molecule resulting in the formation of “diastereoisomeric” co-crystals. Broadly speaking, a supramolecular heterosynthon should favor chirality with respect to homosynthons. This is because a homosynthon is inherently centrosymmetric, while a heterosynthon is inherently non-centrosymmetric and should favor polarity/chirality.

If the chosen coformer is enantiopure, chiral resolution could be achieved via either enantiospecific co-crystal formation, when the co-crystal is formed with only one enantiomer of the target molecule or a diastereomeric co-crystal pair—when the co-crystals are formed with both enantiomers. Unlike for salts, for which diastereomeric salt pair formation appears to be a general rule, co-crystals more frequently behave enantiospecifically. 

The basic idea is that the reaction (whether in solution or in the solid state) of a racemic R,S molecule (R,S-M) with an enantiopure coformer, say an R-coformer (R-C), might lead to the formation of a “diastereoisomeric co-crystal”, namely R-M/R-C and S-M/R-C aggregates. These co-crystals will possess definitely different crystal structures and different physicochemical properties that could be used for resolution. Analogously, if R,S-M is capable of salt formation, say via an acid-base reaction with an enantiopure acid/base capable of hydrogen bonding donor/acceptor interaction via proton transfer, “diastereoisomeric salts” will be formed, e.g., R-MH^+^—R-C^−^ and S-MH^+^—R-C^−^.

**Figure 27:**
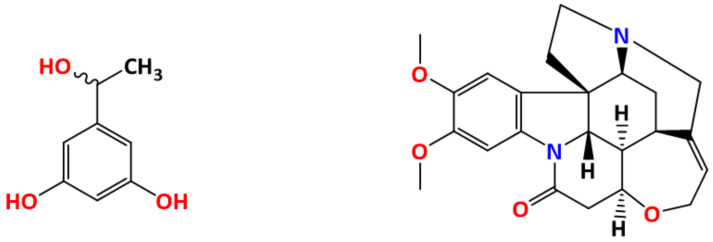
If RS-resorcylmethylcarbinol (**left**) and brucine (**right**) are co-crystallized from methanol, only dextro-resorcylmethyl carbinol forms a co-crystal with brucine [90].

There are several examples in the literature. What is probably the first utilization of the “diastereoisomeric co-crystal” approach dates back to 1939 when Eisenlohr and Meier reported on the chiral resolution of racemic 5-(1-hydroxyethyl)benzene-1,3-diol (resorcylmethylcarbinol) with an alkaloid brucine (see Figure 27).

**Figure 28:**
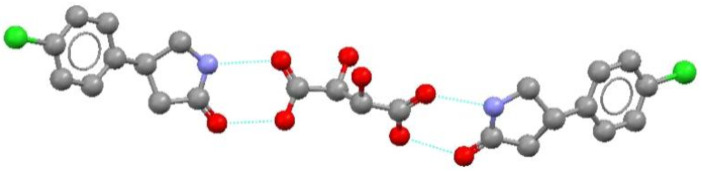
Resolution of optical isomers of 4-amino-p-chlorobutyric acid lactam (Baclofen^®^) by co-crystallization 4-amino-p-chlorobutyric acid lactam lactam [91].

Resolution of optical isomers of 4-amino-p-chlorobutyric acid lactam (Baclofen^®^) by co-crystallization with (2R,3R)-(+)-tartaric acid has been obtained by forming a co-crystal in which only the (R) enantiomer is present. This can be attributed to a supramolecular heterosynthon (see Figure 28) [91].

**Figure 29:**
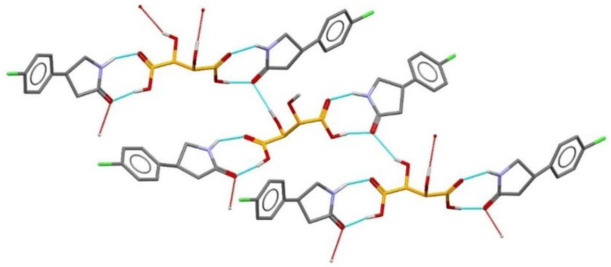
The 2:1 co-crystal of (R-)-4-amino-p-chlorobutyric acid lactam with (2R,3R)-tartaric acid [91].

The unsubstituted (2R,3R)-tartaric acid has been successfully applied for chiral resolution of 4-amino-p-chlorobutyric acid lactam. It was established that only (R-)-4-amino-p-chlorobutyric acid lactam co-crystallizes with (2R,3R)-tartaric acid, resulting in the formation of a 2:1 co-crystal (Figure 29) [91]. 

**Figure 30:**
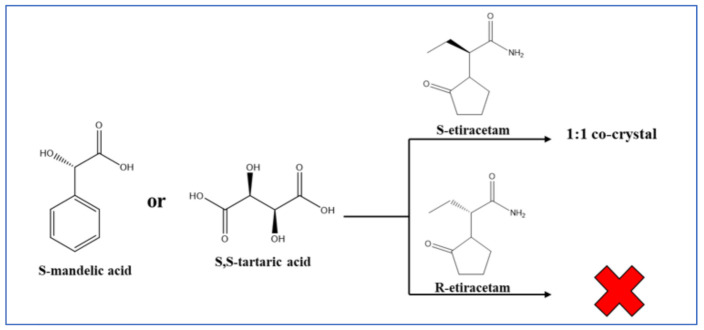
The enantioselective co-crystal formation in the case of S-enantiomer of 2-(2-oxopyrrodin-1-yl)butanamide (S-etiracetam) with S-mandelic and S-tartaric acid. The same reaction with R-etiracetam does not lead to co-crystal formation [92].

Leyssens et al. have shown enantioselective co-crystal formation in the case of 2-(2-oxopyrrodin-1-yl)butanamide (etiracetam) with S-mandelic and S-tartaric acid (see Figure 30), while co-crystals are not formed with the R-etiracetam enantiomer [92]. Conglomerate formation was also reported as a result of crystallization from a racemate via formation of molecular co-crystals [93].

**Figure 31:**
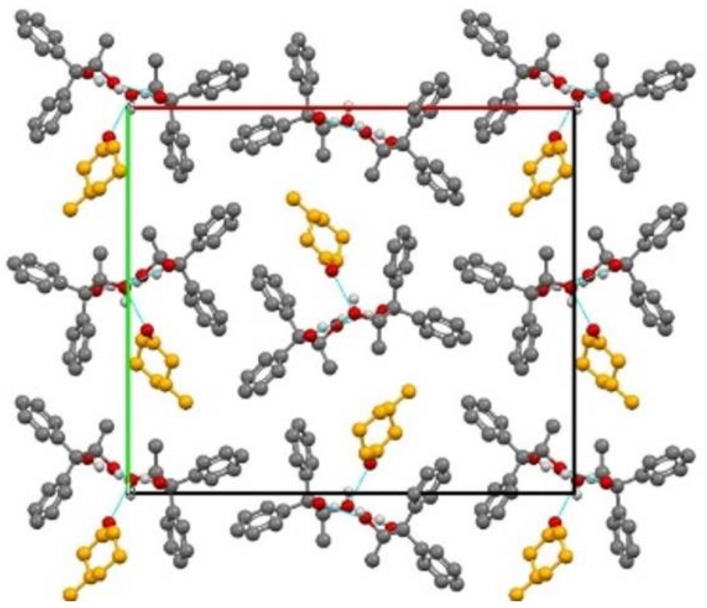
KUCJAT. The carbons of 3-methylcyclohexane are marked in orange and H_CH_ have been removed for the sake of clarity [95].

An alternative route to chiral resolution via crystal engineering is that based on inclusion compounds, a strategy pioneered by F. Toda [94]. Co-crystallization with derivatives of lactic acid led to the enantiomeric excess (ee), including an “inclusion complex” with 3-methylcyclohexanone obtained with an ee > 99% (KUCJAT, Figure 31) [95]. 

A similar approach has been applied for the optical resolution of racemic N-R-3-hydroxypyrrolidines using ((2R,3R)-1,4-dioxaspiro[4.4]nonane-2,3-diyl)bis (diphenylmethanol) as an optically active host compound [96]. Three racemic N-R-3-hydroxypyrrolidine were tested (R=H, benzyl, or ethyl) and it was established that the capability of the optical resolution of ((2R,3R)-1,4-dioxaspiro[4.4]nonane-2,3-diyl)bis(diphenylmethanol) was very high for only N-benzyl-3-hydroxypyrrolidine (ee 100%), whereas for N-ethyl- and 3-hydroxylpyrrolidine, enantioselectivity was low.

## 8. Conclusions 

The purpose of this article was to provide an overview of the main issues concerning drugs in the crystalline state. 

There is little doubt that the design, synthesis, characterization, and biological and pharmacological evaluation of new active ingredients are the first and paramount objectives of research in the pharmaceutical field. However, obtaining a new drug is not enough: the road from bench to market is often long, steep, and tortuous. One aspect that has emerged in all its relevance is that of the impact of the solid-state form of the active ingredient on its utilization as a drug. The vast majority of drugs are constituted of organic molecules. Organic molecules are often structurally non-rigid and conformationally adaptable, and often carry functional groups that form strong and directional intermolecular interactions (hydrogen bonds, halogen bonds, etc.) or that confer anisotropy and polarity, hence impose orientational preference to surrounding molecules, whether of the same or of different type. This variability has consequences when the active ingredient is crystallized from a solution, melt, or vapor phase, because the formation of a solid from a less condensed phase is the outcome of a competition between kinetics and thermodynamic aspects. The isolation of stable or metastable crystal forms, whether enantiotropically or monotropically related, is a manifestation of such dualism, as it is the precipitation of a solvate or of an unsolvated crystal of the API from solution. As we have seen in this review, an active ingredient can be brought in the solid state in an impressive number of alternative forms: solvates, hydrates, salts, co-crystals, and all these forms can be polymorphic. 

These are the reasons why the discovery of a drug, whether destined to treat a new disease or to replace old and less efficacious pharmaceuticals, is undoubtedly a fantastic achievement, but it is also the beginning of a journey in another domain, that of the aggregate form of the new drug, the crystal domain. As a matter of fact, the drug will have to be isolated, its preparation scaled up, purified, stored, packed, distributed, and ultimately administered to patients and all these steps will be carried out, in the vast majority of cases, with the drug in the solid form, sometimes amorphous, most often a polycrystalline material. 

To summarize:

(i) An API can take many solid forms (polymorphs, solvates, salts, co-crystals) all containing exactly the same active principle but in different structural arrangements and/or associated to different molecules (solvents, coformers, counterions, salts, etc.);
(ii) Different solid forms will possess different properties, which might affect a drug’s processing, formulation, distribution, storage, administration to the patient and, ultimately, its therapeutic efficacy;
(iii) Since there is no way to guarantee that a transformation to a more stable form will occur, or that a more stable form will appear, a preliminary, thorough exploration of the crystal domain is the only way to minimize the chances of the undesired appearance of a new and more stable form at a later stage of development;
(iv) Besides maximising control on the crystal form of the API, the investigation of hydrates and co-crystals may afford alternative, often improved or innovative properties of the drug;
(v) Polymorphs, hydrates, and co-crystals may meet the requisites of novelty, utility, and non-obviousness useful for extending the patenting life of a drug.

The examples provided in the article have been chosen with the intention of stressing why the quest for new crystal forms of any given API can be both “joy and sorrow” for the academic and industrial researcher. This article has a provocative title, the same title as the lecture delivered by one of us (D.B.) at the ACCORD22 meeting.

## Figures and Tables

**Table 1:** Physico-chemical properties that may depend on the crystal form.

Physical and Thermodynamic Properties	Density and refractive index, thermal and electrical conductivity, hygroscopicity, melting points, free energy and chemical potential, heat capacity, vapor pressure, solubility, thermal stability, and color and shape of crystals.
Spectroscopic Properties	Electronic, vibrational, and rotational properties, and nuclear magnetic resonance spectral features.
Kinetic Properties	Rate of dissolution, kinetics of solid-state reactions, and kinetic inertness.
Surface Properties	Surface free energy, crystal habit, surface area, and particle size distribution.
Mechanical Properties	Hardness, compression, and thermal expansion.
Chemical Properties	Chemical and photochemical reactivity.

**Table 2:** The five systems used in the sixth blind test.

Target	Chemical Diagram	Crystallization Conditions, Remarks, and Clarifications
(XXII)	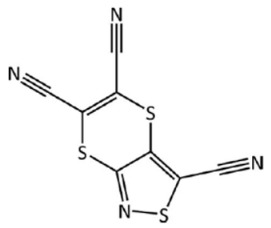	Crystallized from an acetone/water mixture; chiral-like character due to potential flexibility of the six-membered ring, but no chiral precursors used in synthesis.
(XXIII)	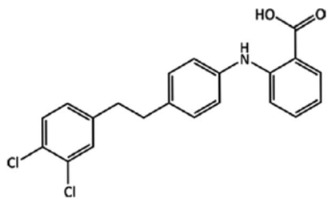	Five known polymorphs (*A*–*E*). The most stable polymorphs at 257 and 293 K. Crystallization conditions include slow evaporation of acetone solution and of an ethyl acetate:water mixture.
(XXIV)	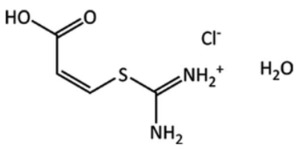	Crystallized from 1 M HCl solution. The substituents of the C=C double bond are in the *cis* configuration.
(XXV)	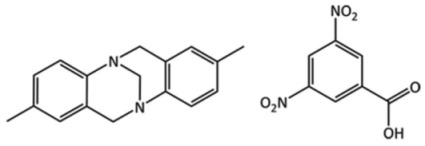	Slow evaporation of a methanol solution, which contained a racemic mixture of the enantiomers of Tröger’s base.
(XXVI)	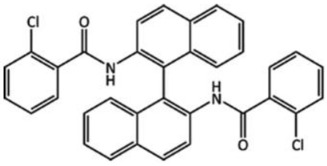	Slow evaporation from 1:1 mixture of hexane and dichloromethane. No chiral precursors used in synthesis.

**Table 3:** Occurrence in the CSD of various crystal forms [56].

CRYSTAL FORMS	% of All Organic Structures
**Organic crystal structures**	100
**Single component molecular organic structures**	72.1
**Single component polymorphic structures**	1.4
**Hydrates**	**7.4**
**Molecular organic hydrates**	2.7
**Polymorphic molecular organic hydrates**	1.0
**Cocrystals**	1.1
**Polymorphic cocrystals**	1.9

**Table 4:** Comparison of solubility data for three examples of hydrated and anhydrous compounds. Data reprinted with permission from Ref. [59]. 2008, Elsevier.

Material	Molecular Structure	Solid State Form	Solubility (mg/mL)
Caffeine (CAF)	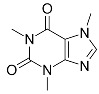	Anh = triclinic β-phase form Hyd = monoclinic 4/5 hydrate	Anh = 49.7 Hyd = 21.8
Carbamazepine (CBZ)	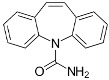	Anh = monoclinic low temp. form Hyd = orthorhombic dihydrate	Anh = 0.424 Hyd = 0.139
Sulfaguanidine (SFG)	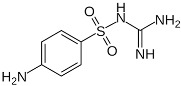	Anh = monoclinic form II Hyd = monoclinic monohydrate	Anh = 1.38 Hyd = 1.07

Rifaximin: An Example of a Multiple Non-Stoichiometric Hydrate.
